# Asymmetric fluttering ferromagnetic bar-driven inertial micropump in microfluidics

**DOI:** 10.1063/1.5017902

**Published:** 2018-02-14

**Authors:** Wonwhi Na, Jinsung Kim, Hoyoon Lee, Byeongmin Yoo, Sehyun Shin

**Affiliations:** 1 Department of Micro/Nano Systems, Korea University, Seoul 02841, South Korea; 2 Department of Mechanical Engineering, Korea University, Seoul 02841, South Korea; 3 Nano-Biofluignostic Research Center, Korea University, Seoul 02841, South Korea

## Abstract

Even though microfluidics has been successfully used in minimizing complicated and onerous processes, the pumping and tubing systems used with it are yet undeveloped and need immediate development. The present study developed a fluttering bar-driven micropump, mounted on a polydimethylsiloxane microfluidic system. The pump consists of a rectangular ferromagnetic bar and a fan-shaped chamber with an inlet and outlet. Through various experiments, the net flow was examined as a function of chamber shape, inlet and outlet channel location, rotating center of the magnet, and rotational speed. Using high-speed camera and image analysis, the net flow was found to be generated by the fluid inertia associated with the varying reciprocating speeds of the bar inside the fan-shaped chamber. Depending on the locations of the inlet and outlet, the cycle time taken to circulate the loop was significantly reduced from 200 to 4 s. The flow rate of the micropump ranges from 48–225 *μ*l/min, which is proportional to the rotational speed of the magnet (150–3000 rpm). Using a fluttering bar-driven inertial micropump, the microfluidic system not only provides improved mixing, but also eliminates certain problems associated with external tubing and connection.

## I. INTRODUCTION

Lab-on-a-chip (LOC) technology has been significantly developed and utilized in diverse applications, including biomedical, fine chemistry, and precision medicine. However, even for such a successful miniaturization, microfluidic systems have suffered from complex plumbing,^1^ fluidic routing,^2^ and difficulties with input/output pumps.^3^ The main cause of these problems is the use of external pumps, which are still the most commonly utilized for LOCs.^4^ Thus, although the issue on the necessity of innovative microfluidic pumping has been raised, the development of a micropump—which is simple, cheap, small, reliable, and easy to integrate to LOC—is still highly demanded.

Diverse micropump technologies and working principles have been introduced in previous reports^5–10^ and these can be classified according to the installation location (internal vs. external pumps) and pumping schemes^11^ (mechanical vs. non-mechanical or passive vs. active pump). For instance, electro-wetting technology^12^ was well developed as a fluid transporting method, which can be integrated with any microfluidic system without any external fluidic connections. This electro-wetting pump is successfully applied to viral diagnostic systems as a commercial product (ePlex, Genmark, CA, USA). A passive capillary pump operating without any external power was also introduced^13,14^ and further developed for a point-of-care immunodiagnostic assay.^15^ However, these sophisticated pumps have not been widely used in LOC because of their limitations such as maximum flow rate and long operating time.

Reciprocating pumps equipped with check valves are suggested,^16^ along with diverse actuation mechanisms such as pneumatic^17,18^ and piezoelectric diaphragms.^19,20^ In fact, reciprocating pumps are commonly available as commercial products and are based on a piezoelectric diaphragm in combination with passive check valves.^21^ However, most of the microfluidic research laboratories use syringe pumps categorized in an external pump. With using these external pumps, there should be a complex fluidic routing and input/output tubing connection. Even though sophisticated LOC studies highlighted the highly integrated inner microfluidic structures with outstanding performance, the complex exterior parts are hidden behind them. Thus, there has been a necessity to develop an internal micropump which can be integrated without a tubing connection.

Recently, there have been noticeable advances in the development of internal micropumps. Yuan and Prosperetti^22^ introduced the concept of a bubble-driven micropump, in which a bubble repeatedly expands and collapses inside a narrow microchannel that asymmetrically connects two large reservoirs. Because of the asymmetric location of the bubble generation in the microchannel between two reservoirs, biased flow inertia is generated in the fluid motion, which results in net flow. Similar inertial pumping employing laser-induced cavitation to create bubbles was further introduced.^23^ Recently, an ink-jet technology was further adopted in bubble-driven micropumps.^24^

All pumps described above are internally installable micropumps having various actuators. However, although these internal micropumps resolve many problems, they do not fully satisfy the requirements. Therefore, the present study introduces a new concept of a fluttering ferromagnetic bar-driven inertial micropump, which can be integrated into a microfluidic system. The key operating principle of the proposed micropump is the asymmetric inertia generation caused by the non-constant fluttering speed of the ferromagnetic bar. In this paper, the operating principle and performance of the present micropump is first covered followed by discussion on the physics behind the pumping mechanism and design optimization.

## II. MATERIALS AND METHODS

### A. Microfluidic chip fabrication

**FIG. 1.**
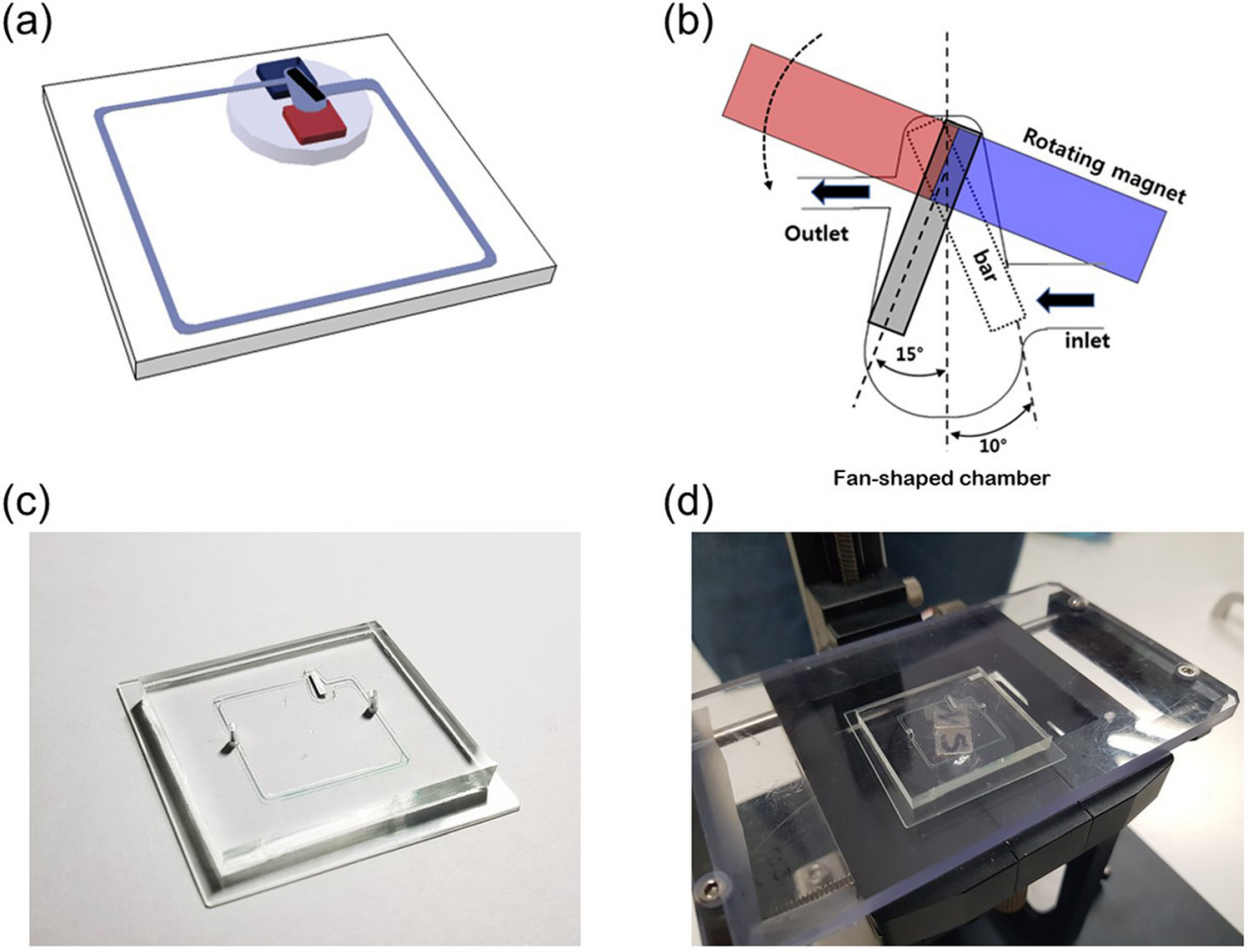
Schematic of the fluttering bar in a fan-shaped chamber: (a) an integrated microfluidic system with a micropump and a closed-loop microchannel, (b) detailed view of a micropump consisting of a rotating magnet, a fluttering ferromagnetic bar, and a fan-shaped chamber with an inlet and an outlet, (c) a photographic image of the microfluidic chip, and (d) the experimental setup of the micro pump system.

The microfluidic chip, as shown Fig. 1(a), is made of polydimethylsiloxane (PDMS), and consists of a microchannel and a fan-shaped chamber. The PDMS chip, 40 mm × 40 mm × 5 mm, was designed by a 3D modeling tool (Catia, Dassault systems, Vélizy-Villacoublay, France). The width of the microchannel is either 700 or 1500 *μ*m, depending on its location, and the channel height is 180 *μ*m. A cutter plotter (Craft ROBO, Graphtec, CA, USA) was used to pattern the micropump chamber, its inlet and outlet, and the microchannel on an adhesive tape. The patterned microstructure was attached on a square dish. Afterwards, the liquid PDMS (Sylgard 184a) silicone elastomer and curing agent (Sylgard 184b) mixed in a 10:1 weight ratio, (Dow Corning Corp.) were poured onto the microstructure attached on the square dish. The square dish was degassed by a desiccator for 30 min to remove any air bubbles present in the PDMS and cured in a dry oven (70 °C) for 6 h. After the curing process, the microstructure-engraved PDMS was peeled from the square dish. Two holes, as sample inlet and outlet, are punched on the microchannel. The PDMS layer and glass substrate was treated by O_2_ plasma (Covance, FEMTO SCIENCE, Yongin, Korea) for 50 s to activate the surfaces. The ferromagnetic bar [5 mm (l) × 1 mm (w) × 160 *μ*m (h)]—made of a ferromagnetic material (SUS430) and coated by the sputtering method with TaN (tantalum nitride) with a thickness of 300 nm on the surface to prevent rusting—was placed inside the magnetic micropump chamber. The upper PDMS and lower glass substrate layers were combined, and for a stronger bond between them, this microfluidic chip was placed in a dry oven (60 °C) for 2 h. Accordingly, a microfluidic chip containing a closed-loop microchannel, micropump chamber, and ferromagnetic bar was fabricated.

### B. Configuration of the micropump driving system

The fluttering bar-driven micropump was applied to a microfluidic closed loop, as shown in Fig. 1(a). A closed loop is frequently used for performing polymerase chain reactions. The dimensions of the channel are 700 and 180 *μ*m in width and depth, respectively. However, through design optimization, the width of the inlet channel is determined as 1500 *μ*m. The dimensions of the fluidic chip are 26 mm × 26 mm and having an internal volume of 15 *μ*l.

The micro pump driving system consists of two parts—a motor module for closed-loop circulation, and x, y, and z stages. The motor module, located at the bottom of the device, consists of a motor (EC-I, Maxon motor, Sachseln, Switzerland) precisely controlled by a computer and a magnet mount. The magnet mount has two neodymium magnets—one with the N-pole up and the other with the S-pole up (Kingkong magnet, Seoul, Korea). The microfluidic chip is mounted on the x, y, and z stages and can be precisely positioned in the x, y, and z directions on the stage to optimize pumping performance.

Figure 1(b) presents a schematic of a fluttering ferromagnetic bar inside a chamber with an inlet and outlet. The chamber is fan-shaped with an arc subtended by a 20° angle. The pump inlet and outlet are located radially outward and inward, respectively. In addition, the inlet width is twice the outlet width. All these design parameters have been optimized through experimental and numerical simulations. A detailed description is given in Sec. III. The bar is a rectangular strip, remotely driven by a rotating magnet mounted on the electric motor shaft. The depth of the chamber and height of the bar are 180 and 160 *μ*m, respectively. Because the rotation center of the bar is located at one sixth of the bar reckoned from the top, its rotation angle is 30°, which is greater than the angle of the fan-shaped chamber (20°).

### C. Flow visualization and high speed camera analysis

The pumping mechanism and performance were analyzed by flow visualization. Small bubbles (∼150 *μ*m) were injected into the microchannel to visualize the net flow per pumping cycle. Fluorescence microbeads (15 *μ*m, $\lambda_{Ex}$: 468 nm, $\lambda_{Em}$: 508 nm) were also introduced to observe the vortices generated inside the pumping chamber. The displacements of the bubbles, caused by the flow in the discharge and suction modes of a pumping cycle, and streamline of the moving fluorescence microbeads were observed by a high-speed camera (FASTCAM, Photron, CA, USA). The rotating speeds of the ferromagnetic bar during the discharge and suction modes with varying rpm in the external magnet rotation were similarly observed using a high-speed camera. Finally, a one-turn rotation time was analyzed using colored dye injected into the microchannel. The micropump was then operated until the dye circulated through the entire closed-loop.

## III. OPERATING PRINCIPLES AND METHODS

### A. Inertial micropump using a fluttering ferromagnetic bar

As the magnet rotates counter-clockwise (CCW), the ferromagnetic bar rotates in the same direction. However, because motion is prevented by the fan-shaped chamber, the bar cannot sweep through the full angle of the rotating magnet and stops, as shown Fig. 1. Then, as the rotating magnet continues its rotation, the bar suddenly rapidly rotates in the clockwise (CW) direction. The clockwise rotation of the bar is because of the sudden change in the magnetic field inside the fan-shaped chamber associated with the rotating magnet. Interestingly, this clockwise rotation is not only opposite to that of the rotating magnet, but also considerably faster. These results are because of the changes in the magnetic field associated with the rotating magnet, as discussed in Sec. III B. Thus, there are two types of bar rotations inside the fan-shaped chamber: one is the relatively slow CCW rotation following the rotating magnet and the other is the fast CW rotation corresponding to the change in the magnetic field.

**FIG. 2.**
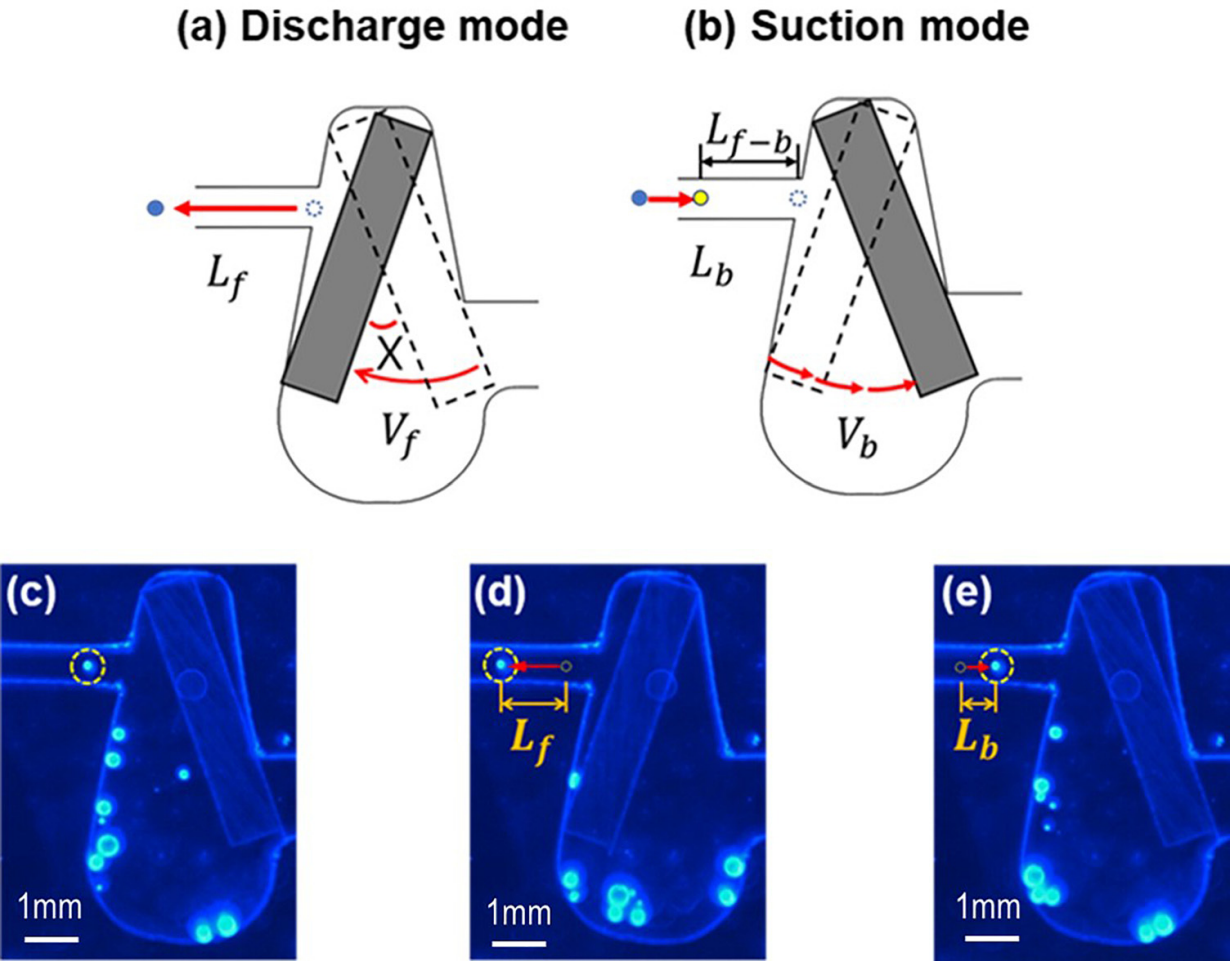
Operating principle of the fluttering bar-driven inertial micropump: (a) discharge mode, (b) suction mode, (c) initial state*, (d) after discharge mode*, and (e) after suction mode*. *Multimedia view: https://doi.org/10.1063/1.5017902.1
10.1063/1.5017902.1

When a bar rotates, the fluid near the bar gains inertia, which is proportional to the rotational speed of the bar. As described above, the change in the rotational speed of the bar results in a change in fluid inertia. As shown in Fig. 2(a), when the bar is rapidly rotated clockwise, the fluid near the outlet gains a large amount of inertia and yields a large forward displacement (*L_f_*) during the discharge mode. Conversely, when the bar slowly rotates counter-clockwise, the fluid exiting the outlet has a relatively small inertia and yields a small backward displacement (*L_b_*) during the suction mode, as shown in Fig. 2(b). Subsequently, the difference in inertia between two directional flows leads to a non-zero displacement (*L_f-b_*) in the forward direction.

The explanation of the operating principle of the present micropump described above was verified using flow visualization, as shown in Figs. 2(c)–2(e) (Multimedia view). Prior to the rapid rotation of the bar, there is a fluid particle near the exit of the micropump, as shown in Fig. 2(c). When the bar rapidly rotates, the particle is displaced to the left through a distance, *L_f_*, which is between the particle positions shown in Figs. 2(c) and 2(d). When the bar slowly rotates in the CCW direction, the exiting fluid particle is drawn back through a relatively small displacement, *L_b_*, which is between the particle positions shown in Figs. 2(d) and 2(e). Hence, the particle would have a non-zero displacement (*L_f-b_*) in the forward direction, resulting in a net flow rate. This result means that the fluttering bar, having unequal rotational speeds, provides the fluid with net inertia and thus acts as a pump to create a net flow rate. It is noteworthy that if the bar rotates at equal speeds, then there would be no net flow rate.

### B. Rotating magnetic field analysis

**FIG. 3.**
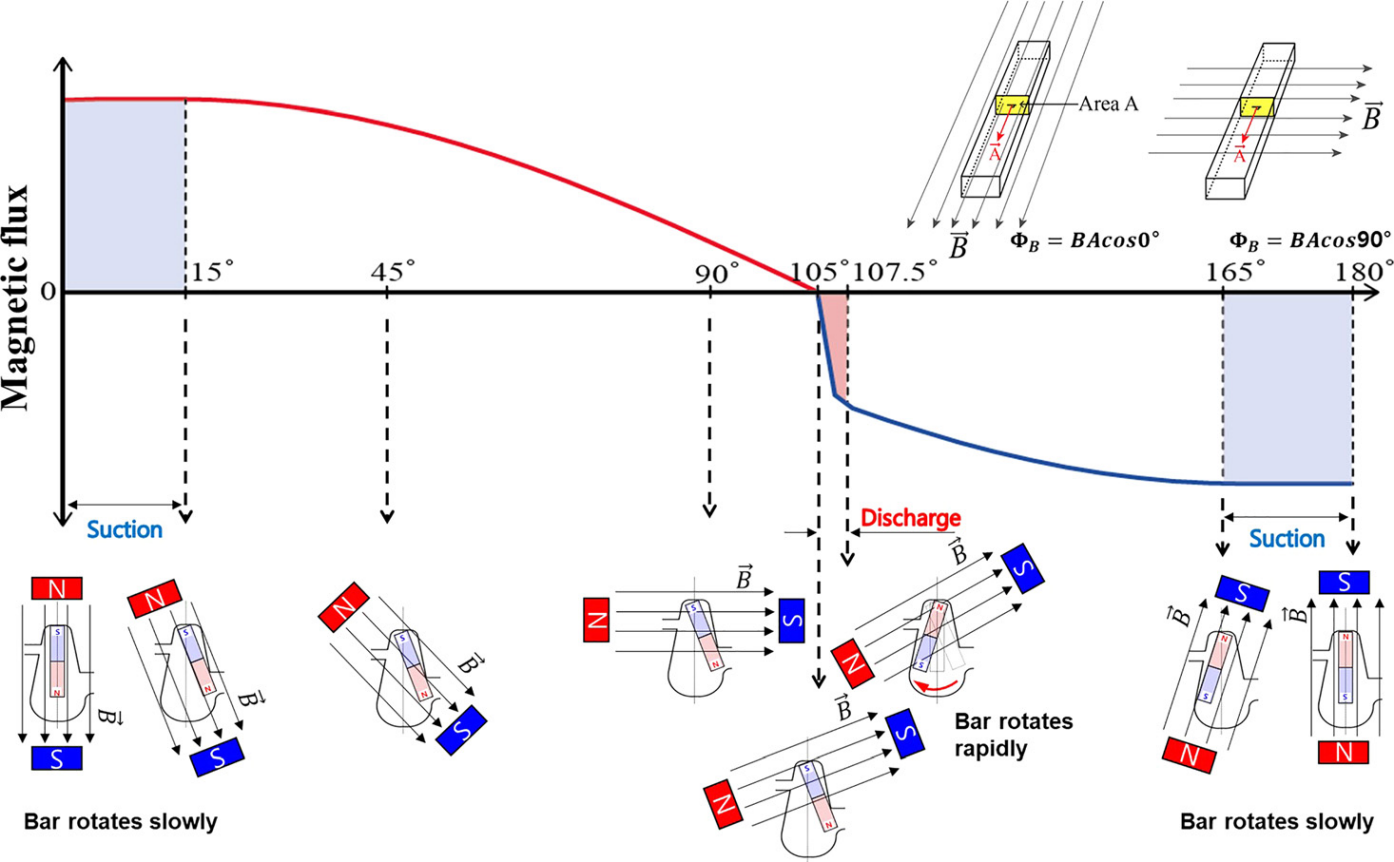
Magnetic field analysis for a fluttering bar in a fan-shaped chamber while an external magnet rotates 0°–180°.

As described above, the net flow rate was generated by the different angular speeds of the ferromagnetic bar between forward and backward directions. The difference in angular speed is because of the variations in the magnetic field inside the fan-shaped chamber, as shown in Fig. 2. As the magnet rotates in the CCW direction, the corresponding magnetic flux passing through the cross section of the ferromagnetic bar (area A) of the bar inside the chamber is not that easily predicted, as shown in Fig. 3, Fig. S1 in the supplementary material. The bar trapped inside the chamber could not follow the rotating magnet but limited to within 30°. If the chamber is a full circular one, then the center of rotation will be the same as the geometric center of the chamber and the bar would rotate as the rotating magnet, and magnetic flux passing through area A is a constant maximum value in all magnet rotation angles. However, because of the pie-shape of the chamber, the magnetic flux passing through area A of the bar yields the distorted sine wave shown according to the changes effected by the rotating magnetic field.

As shown in Fig. 3, as the S-pole of the rotating magnet passes 0°, the magnetic flux passing through area A yields the maximum plateau within the angle of the bar rotation (from 0° to 15°). Then, the magnetic flux gradually decreases until the magnet rotates to 105°. As the magnet further rotates, the N-pole of the rotating magnet gets closer to the bar than the S-pole. Accordingly, the polarity at the lower end of the magnetized ferromagnetic bar suddenly changes from the N-pole to S-pole. Simultaneously, the bar end yielding to the S-pole rapidly rotates to the approaching magnet with the N-pole end. This movement occurs within a very short duration and through a small angle (105°–107.5°). It is noteworthy that the rotation is opposite to that of the rotating magnet. Furthermore, using a high-speed camera, we confirmed that the bar rotation is faster than that of the rotating magnet. This fast rotation in an opposite direction is mainly because of the sudden change in the magnetic flux direction passing through the bar trapped inside the fan-shaped chamber.

Then, the bar stops until the N-pole end of the magnet approaches. Again, the bar rotates with the rotating magnet within the angle range of 165°–180° and stops at 195° (Fig. S2, supplementary material). Figure 3 shows the half-cyclic analysis of the magnetic flux passing through area A for a bar trapped inside the fan-shaped chamber. The second half-cycle is simply based on symmetry.

Interestingly, when a sudden change in the magnetic polar field occurs, the bar in the chamber showed a significantly fast rotation, which acts as a discharge mode of the pump. Even though this rotation is completed within a very short time (Δt = 5 ms), the pumping mode is effective for a while because of fluid inertia. The fast angular velocity of the bar generates a strong inertia in the corresponding direction, causing a large displacement of the particle and vice versa, as shown in Fig. 2. Thus, the abrupt change in the magnetic field of the bar results in a difference in the fluid inertia between the discharge and suction modes, and subsequently leads to a non-zero flow rate through the use of the fluttering bar. Evidently, this interesting variation in the magnetic field was generated with the combination of a rotating magnet and a ferromagnetic bar trapped inside the fan-shaped chamber.

## IV. RESULTS AND DISCUSSION

**FIG. 4.**
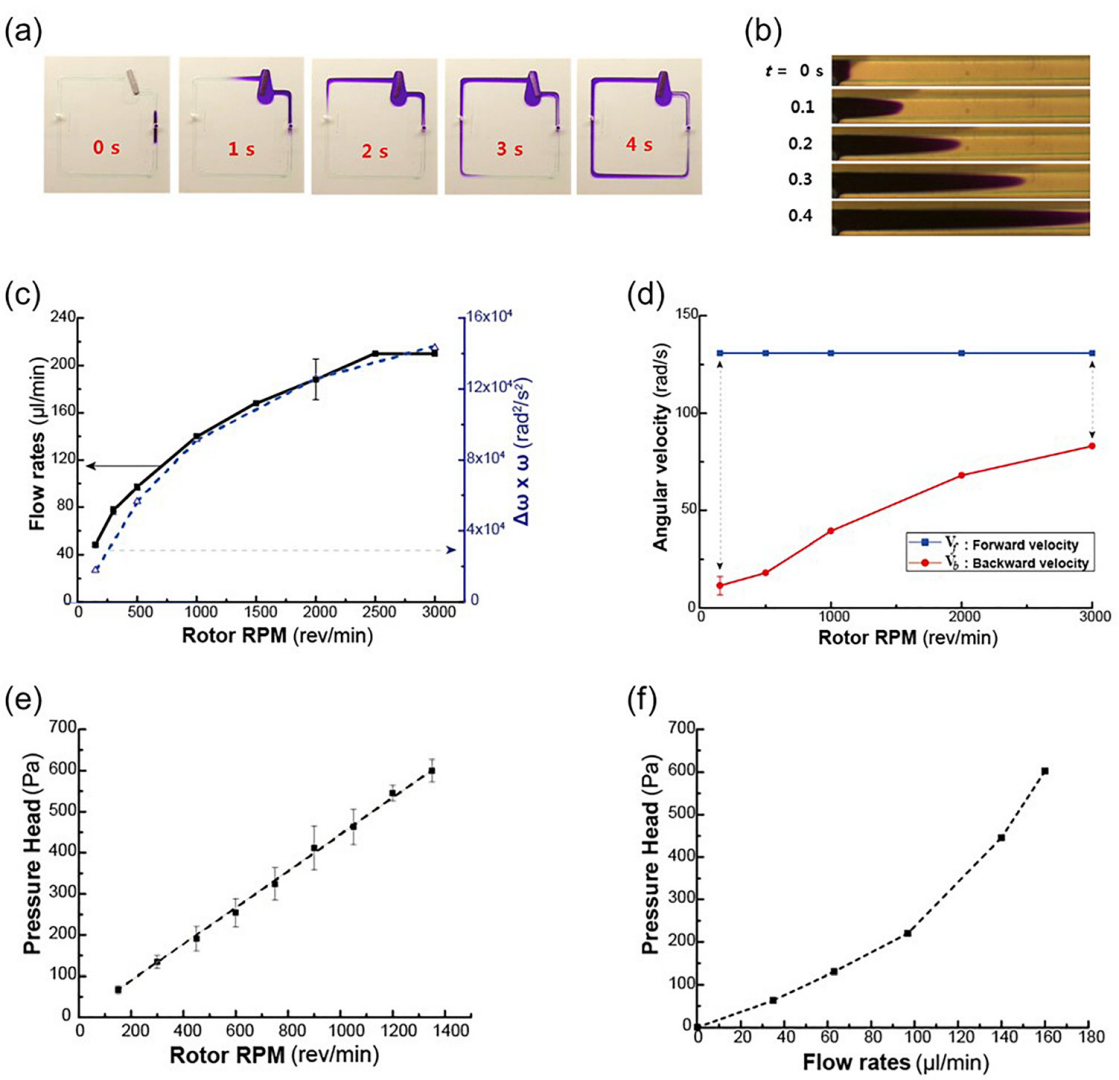
(a) Visualization of pumping flow with the injected dye, (b) visualization of Taylor-dispersion in a microchannel, (c) pumping performance curve with flow rate vs. rotor rpm, (d) angular velocity of a bar in CW and CCW-directions, (e) pressure head vs. rotor rpm, and (f) the pressure-flow rate curve. Multimedia view: https://doi.org/10.1063/1.5017902.2
10.1063/1.5017902.2

As the bar begins to flutter, the injected dye circulates with the fluid and completes a cyclic motion within 4 s, as shown Fig. 4(a) (Multimedia view). As the dye is continuously injected, a purple-color flow is clearly visible. The fluttering bar makes the injected dye circulate through a closed loop within 4 s when the magnet rotates at 3000 rpm. Also, the flow generated by the fluttering pump yields a parabolic velocity profile. Also, we observed the mixing effect of the fluttering ferromagnetic bar (supplementary material, Video1.wmv). Many vortices generated in fan-shaped chambers in different directions during discharge and suction modes, effectively mix the liquid.

When the dye is injected into the parabolic velocity field, a diffusion phenomenon with a long tail is observed in Fig. 4(b). Because the dye is continuously injected, the tail of the Taylor dispersion is not observable, except for the head. The diffusion is mainly because of the laminar velocity-driven convective diffusion, which is frequently observed in a laminar-flow microfluidic channel. The parabolic velocity profile in the microfluidic channel makes fluid velocity difference between the channel wall and the channel center.

Pumping performance was analyzed with the flow rate function of the rotor speed in rpm. The minimum flow rate obtained was 48 *μ*l/min at a rotor speed of 300 rpm. As the rotor speed increases, the flow rate increases and plateaus at 225 *μ*l/min when the rotor speed is 2500 rpm. Furthermore, the pumping flow rate does not linearly increase with the rotor rpm, as shown in Fig. 4(c). The performance curve yields a square root curve and plateaus at 2500 rpm. The non-linear performance curve is because of the characteristic of the inertial pump. As the rotor speed increases, fluid inertia correspondingly increases.

However, as shown in Fig. 4(d), as the rotor speed increases, the CCW-direction speed of the bar (red line) increases, whereas the CW-direction speed of the bar (blue line) remains nearly constant. Thus, the difference between the speeds in the two directions gradually decreases with the rotor speed and as a result, the difference between the inertias in the two directions gradually decreases. Consequently, the product of the speed difference (Δω) and the rotor speed (ω) yields a curve similar to that of the volume flow rate in Fig. 4(d).

Also, we measured the hydrostatic pressure by mounting a capillary tube at the right after the pump outlet. By calibrating the surface tension effect on the capillary, the static pressure was measured by increasing the rotor speed. Considering dynamic pressure, the total pressure head can be obtained and plotted against the rotor rpm in Fig. 4(e). The pressure head linearly increases with the rotor speed. In addition, combining the total pressure head and the flow rate, we obtained a curve of pressure head vs. flow rate, as shown in Fig. 4(f). The pressure-flow rate curve yields a parabolic shape, which is the same trend of a system curve of a pump. If we change either the dimensions of a microchannel or the viscosity of the working fluid, we can obtain a set of system curves.

**FIG. 5.**
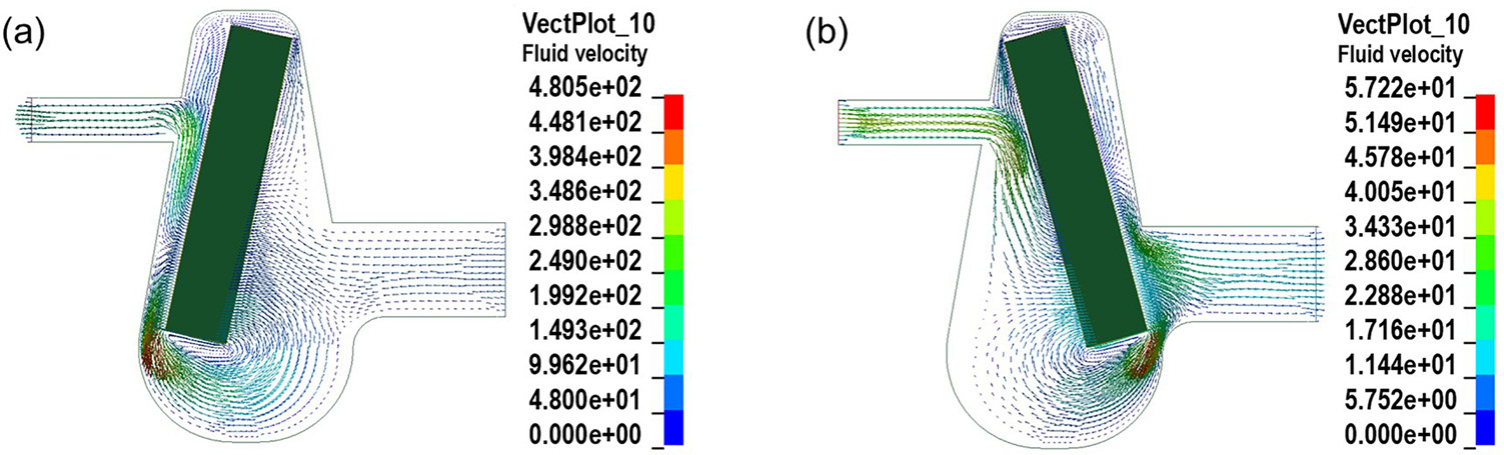
Numerical simulation of the fluttering bar-driven micropump: (a) velocity plot at the discharge mode and (b) velocity plot at the suction mode.

Numerical simulation was performed with LS-DYNA (KOSTECH, Seoul, Korea). The numerical results support the present experimental results, as shown in Fig. 5. When the bar in the fan-shaped chamber rapidly rotates in the CW-direction, then the maximum velocity at the pump outlet is about 0.19 m/s in Fig. 5(a), which is almost similar to the value (0.21 m/s) obtained by the high speed camera, as shown in Fig. 2(d). When the bar slowly rotates in the CCW-direction, a part of the discharged fluid flows back to the chamber, some of which is discharged through the inlet channel, as shown in Fig. 5(b). The maximum velocity at the pump outlet in Fig. 5(b) is about 40 mm/s, which is a little higher than the experimental one (50 m/s). The difference in velocities could be caused by 2-dimensional assumption in the numerical analysis for calculation simplicity.

**FIG. 6.**
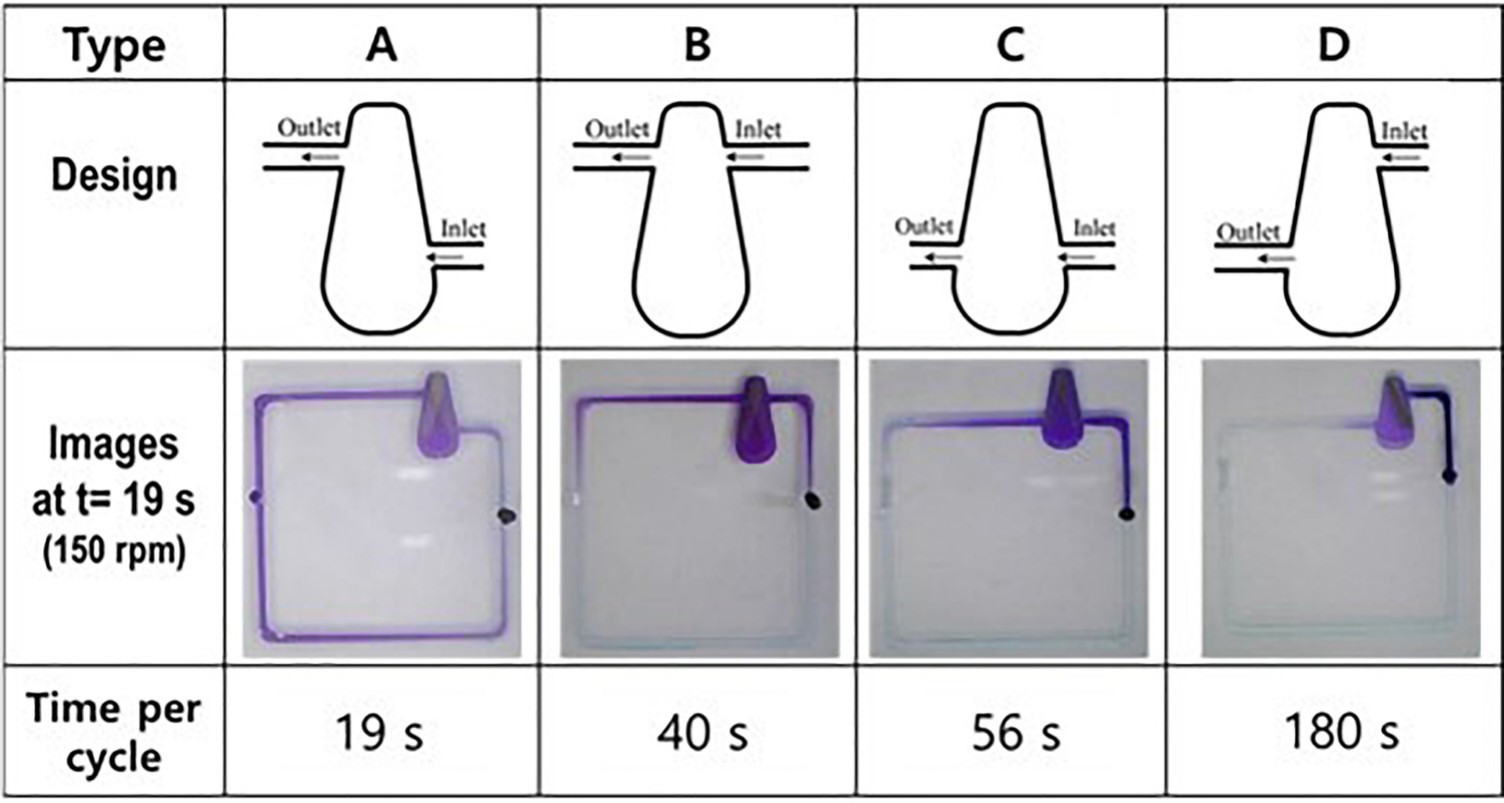
Comparison of various designs for optimization corresponding to various locations of the inlet and outlet in a fixed fan-shaped chamber. Multimedia view: https://doi.org/10.1063/1.5017902.3
10.1063/1.5017902.3

We also examined the effect of the inlet and outlet positions on pumping performance in terms of the time required to circulate the fluid through a cycle, as shown in Fig. 6 (Multimedia view). We examined four different designs having different positions of inlet and outlet channels. Unexpectedly, the locations of the inlet and outlet channels performed important roles in the pumping efficiency. In the same figure, type A—exhibiting the fastest cycle time of 19 s—is used as the reference. When both the inlet and outlet channels are near the center of rotation (type B condition), the circulation time is 40 s, which is more than twice the reference value. In the case of type C, where the inlet and outlet channels are located at the same outer radii from the center, the circulation time is 56 s. In type D, the inlet and outlet are located at the inner and outer radii, respectively, and the circulation time significantly increases to over 180 s.

Comparing types A and B would lead to a clear understanding of the mechanism. The difference between A and B is the inlet location. Because the inlet of type A is located at the outer radius, this inlet would strongly suck the fluid because of inertia. Comparing types B and D, the locations of the outlets are different, and the type in which the outlet is located at the outer radius (type D) yields less effective pumping. This fact implies that the outlet would be better located at the inner radius to effectively trap the fluid and squeeze it through the outlet. In other words, pumping involves the combination of the driving fluid into some limited space and adding inertia to force it out. In that sense, the rapidly rotating bar drives the fluid from the outer radius toward the inner radius and closes the pathway to prevent its escape, except through the outlet. Hence, the most efficient pumping performance was observed under type A, rather than the other designs.

The pump with an optimal design has been operated at various rotor rpm and the generated flow rates and pressures were analyzed in Fig. 4. However, it would be better to compare the performance metrics of the present pump to those of the existing pumps. Compared to a commercial piezoelectric pump used in a similar scale of a microfluidic system,^19^ the results are as follows: the present inertia pump operating at 2.5–50 Hz (150–3000 rpm) produces the flow rate of 40–210 *μ*l/min, whereas the piezo-pump operating at 60 to 110 Hz produces a flow rate of 160 *μ*l/min. The internal volume of the pump chamber is 4.32 mm^3^ for the present pump and 15.7 mm^3^ for the piezo-pump (d = 10 mm, h = 0.2 mm), respectively. Therefore, the present inertial pump has better pumping performance per unit volume, per unit pulse than the commercial piezo-pump.

## V. CONCLUSIONS

We introduced an inertial micropump consisting of a fluttering ferromagnetic bar in a chamber. Throughout the present study, pumping was mainly caused by the asymmetric inertia associated with the non-equal reciprocating speeds of the fluttering bar. The difference in rotating speeds was also induced by the sudden change in the magnetic field inside the confined fan-shaped chamber with a rotating magnet. Consequently, because the micropump utilized the varying rotational speeds in the two directions, the pump belongs to the category of inertial micropumps. The main feature of the present inertial micropump is its potential integration with any microfluidic system, eliminating all tubings and connections for pumping. In addition, the present micropump provides efficient mixing through the fluttering of the bar inside the fan-shaped chamber, thus not requiring any additional configuration in the mixing device. With further improvements in the rotational magnet driving mechanism with miniaturized electric magnetics, the present micropump can be applied to a wide variety of microfluidic systems.

## VI. SUPPLEMENTARY MATERIAL

See supplementary material for Fig. S1 which shows an analysis of magnetic flux for a ferromagnetic bar with a rotating magnetic field and Fig. S2 which shows a magnetic field analysis for a fluttering bar in a fan-shaped chamber while an external magnet rotates from 0° to 360°.
